# Efficacy and Mechanism of Electroacupuncture Treatment of Rabbits With Different Degrees of Knee Osteoarthritis: A Study Based on Synovial Innate Immune Response

**DOI:** 10.3389/fphys.2021.642178

**Published:** 2021-08-05

**Authors:** Anmin Ruan, Qingfu Wang, Yufeng Ma, Dong Zhang, Lili Yang, Zhongpeng Wang, Qi Xie, Yueshan Yin

**Affiliations:** ^1^Department of Orthopedics, Beijing Longfu Hospital, Beijing, China; ^2^Department of Tendon and Injury, The Third Hospital Affiliated to Beijing University of Chinese Medicine, Beijing, China; ^3^Department of General Surgery, The Second Hospital Affiliated to Beijing University of Chinese Medicine, Beijing, China; ^4^Acupuncture and Moxibustion Department, The Third Hospital Affiliated to Beijing University of Chinese Medicine, Beijing, China; ^5^Graduate School, Beijing University of Chinese Medicine, Beijing, China

**Keywords:** electro-acupuncture, knee osteoarthritis, innate immune response, signaling pathway, mechanism research

## Abstract

Knee osteoarthritis (KOA) is a chronic degenerative bone and joint disease, which is often clinically manifested as pain, joint swelling, and deformity. Its pathological manifestations are mainly synovial inflammation and cartilage degeneration. This study aims to investigate the efficacy of electro-acupuncture (EA) on model rabbits with varying degrees of KOA and to study the mechanism of EA on KOA based on the innate immune response. Mild and moderate rabbit KOA models were established using a modified Hluth method, and EA was given to both the mild and moderate model groups. The Lequesne-MG index was used to evaluate the behavioral changes in the rabbits before and after EA treatment. Morphological changes in the synovial membrane and cartilage of each group were observed by H&E staining. The Mankin scoring standard and the Krenn scoring standard were used to score the pathology of the cartilage tissue and synovial tissue, respectively. The inflammatory factors and metalloproteinases were detected in the serum of each group by ELISA. The protein and messenger RNA (mRNA) expressions of important elements related to Toll-like receptors (TLRs)-mediated innate immune response in the synovial tissue were detected by Western blot and quantitative PCR (qPCR). The Lequesne-MG index score of the rabbits gradually increased with the modeling prolonged but decreased significantly after EA treatment, indicating that EA has a better effect on alleviating the pain and improving the dysfunction. The morphological analysis showed that the inflammation of and the damage to the synovial membrane and the cartilage tissue gradually deteriorated with the modeling prolonged. However, the synovial membrane inflammation was significantly relieved after EA treatment, and the cartilage injury showed signs of repair. The ELISA analysis showed that, with the modeling prolonged, the serum-related inflammatory factors and mechanism of metalloproteinases gradually increased but decreased after EA treatment. The tumor necrosis factor alpha (TNF-α), interleukin 6 (IL-6), and matrix metalloproteinase3 (MMP3) of EA1 group were significantly lower than those of EA2 group. Both Western blot and qPCR results showed that the protein and mRNA expressions of the elements related to the innate immune response in the synovial membrane increased gradually with the modeling prolonged, but decreased significantly after EA treatment. Additionally, the expression of some components in EA1 group was significantly lower than that in EA2 group. These results confirm that synovial inflammation gradually aggravated with time from the early to mid-stage of KOA. EA alleviated the inflammation and histological changes in KOA rabbits by inhibiting the TLRs-mediated innate synovial immune response. This suggests that using EA in the early stage of KOA may achieve a desirable efficacy.

## Introduction

Knee osteoarthritis (KOA) is a clinically common degenerative musculoskeletal disease that involves the cartilage, the subchondral bone, the synovium, the joint capsule, and other joint structures. It is characterized by all-round, multi-level, and varying degrees of chronic inflammation (Atukorala et al., 2016; Wang et al., 2018; Hawker, 2019). At present, it is generally believed to be one of the main causes of disability. Its incidence worldwide is close to 4%, with more than 35% of middle-aged people affected by it. Its high incidence brings heavy economic burdens to its victims and their family (Cross et al., 2014; Thomas et al., 2014). Currently, the recommended therapeutic options for KOA include pharmacological, non-pharmacological, and surgical interventions.

However, up to now, there is no effective method that can completely cure KOA, and the only effective method to treat pains in the late stage is knee replacement (Deyle et al., 2020). Due to individual, family, and social factors, knee surgery is unlikely to become a universal treatment. Although non-steroidal anti-inflammatory drugs (NSAIDs) are currently a preferred treatment for KOA, their long-term use can cause serious adverse reactions in some patients. Therefore, more and more clinicians and patients are looking forward to non-drug interventive therapies. Among them is electro-acupuncture (EA). EA, commonly used in the treatment of osteoarthritis (OA) worldwide, can effectively relieve pain, joint stiffness, and KOA-caused dysfunction. It has the advantages of quick onset, good long-term efficacy, low adverse reactions, simplicity, safety, and economy, to name just a few (Barnes et al., 2008; Ahsin et al., 2009). EA, combining traditional acupuncture with electrical stimulation, in which a small electric current is passed between pairs of acupuncture needles, can increase the efficacy of acupuncture compared with traditional acupuncture. However, its mechanism in KOA treatment remains to be known and needs further study.

Previous studies reported that cartilage injury is the cause and result of KOA lesions. With more studies on it, synovitis has been gradually recognized to play an important role in the clinical manifestations and pathological development of KOA (Berenbaum, 2013). During its disease development, synovitis of joints in many patients with OA is characterized by non-infectious chronic inflammation, but its cause is still controversial at present (Sellam and Berenbaum, 2010). The theory of synovial innate immune response has become a relatively well-recognized mechanism. Namely, with the degeneration of the joint, the damaged extracellular matrix and the shed debris enter the joint cavity, stimulate the synovial membrane to initiate the innate immune response, and promote the inflammatory mediators to release into the synovial fluid to affect the cartilage and the entire joint cavity. Toll-like receptors (TLRs), an important type of pattern recognition receptors (PRRs) in the innate immune system, have been found by current studies to be expressed in the OA articular cartilage and the synovial cells and almost always detected in normal synovial tissues. For example, Radstake et al. showed that although the expression of TLRs could hardly be detected in normal synovial tissues, TLR2 and TLR4 in TLRs were highly expressed in the OA synovial tissues (Goldring and Otero, 2011; Sokolove and Lepus, 2013; van den Bosch, 2019). This suggests that the TLRs-formed signaling pathway is crucially involved in the pathological process of KOA synovitis. However, whether EA can treat KOA by regulating TLRs-mediated innate immune response is exactly what we need to verify. In view of this, based on the animal model, this study applied electrical intervention to varying degrees of KOA and used the improved Lequesnemg index score to record the behavior of rabbits in each group before and after EA treatment. The synovial membrane and cartilage before and after EA treatment were observed by histological method, and the serum and synovial tissue of the rabbits in each group was assessed by molecular biology technique to explore the effect of EA on TLRs-mediated inherent immune responses in knee OA synovial inflammation and to further clarify the mechanism of action of EA treatment of KOA.

## Materials and Methods

### Instruments and Reagents

Instruments needed are disposable acupuncture needles, 0.25 mm × 25 mm (Dongbang, Suzhou, China) and INTI pulse acupuncture treatment instrument, Model: KWD-808I (Yingdi, Changzhou, China). Reagents acquired are rabbit matrix metalloproteinase 1 (MMP1), MMP3, MMP13, tumor necrosis factor alpha (TNF-α), interleukin 6 (IL-6), and interleukin 1 beta (IL-1β) enzyme-linked immunoassay kit (Abcam, United States); TLR4, nuclear factor-kappa B (NF-κB), MyD88, TLR2, and TRAF6 monoclonal antibodies (Abcam, United States); fluorescence Quantitative PCR instrument (Applied Biosystems, United States); ECL Luminometer (Millipore, United States); TRNzol total RNA extraction kit (TIANGEN, Beijing, China); PCR test kit (Nuoweizhan, Nanjing, China); PCR primer synthesized (MDL, United States); and BCA protein kit (Thermo, United States).

### Animal Model Establishment

Forty-eight healthy, 6-months-old New Zealand white rabbits purchased from Beijing Jinmuyang Experimental Animal Breeding (Beijing, China), with 24 male and 24 female weighing 2.0–2.5 kg each, were randomized into six groups, including the blank group (B), the sham operation group (S), model 1 group (M1), model 2 group (M1), the electroacupuncture group 1 (EA1), and the electroacupuncture group 2 (EA2). With eight rabbits in each group, all rabbits were reared in a clean cage at room temperature (22 ± 1°C) and with the air humidity at 40–50%. They were fed with conventional pellets, with drinking water given. The environment was quiet and comfortable, and single-cage feeding was conducted. After 1 week of adaptive feeding, the improved Hulth method (Rogart et al., 1999) was used to prepare a single knee joint OA model. After anesthesia with sodium pentobarbital solution (3% concentration, 1 ml/kg) was injected, surgical modeling was performed on the skin for the left knee joint. The incision was sutured layer by layer with absorbable surgical sutures after the joint cavity was fully rinsed with normal saline and iodine complexation and the wound was covered with sterile dressing. After the operation, the affected limb was not fixed and the animals were driven away to shorten the modeling process. The modeling of the M1 and EA1 groups lasted 4W, and the modeling of M2 and EA2 group lasted 8W. Dressing the change and intramuscular injection of gentamicin injection (0.125 ml/kg) were performed once a day to prevent postoperative wound infection.

The blank group was fed under the same conditions without surgical treatment. The sham-operated group was cut open at the joint capsule by the same approach and sutured layer by layer after irrigating with normal saline to exclude the influence of surgery on the experimental results. The experiment was approved by the Ethics Committee of Animal Experiment of Beijing University of Chinese Medicine, Beijing, China, to minimize the number of animals used and to minimize the suffering of the animals.

### Treatment

Rabbits of Group B, Group S, Group M1, and Group M2 were fed normally without any treatment. Acupoints in the EA 1 and the EA 2 groups were selected according to the human acupoints, and the animal acupoint analogy method was described in “Experimental Acupuncture” and “Animal Acupuncture,” respectively. Acupoints “Ex-LE4,” “ST-35,” “GB34,” “SP9,” “SP10,” and “ST34” were selected to perform acupuncture with filamentous needles. Oblique pricks “Ex-LE4” and “ST-35” and other acupoints are directly pricked about 5-mm deep hole. Intipulse acupuncture apparatus was used for EA treatment. One group of EA connections was “positive electrode: ST34–negative electrode: SP10,” and the other group was “positive electrode: ST-35–negative electrode: Ex-LE4.” The intensity of density wave was 1–2 mA for 20 min each time according to the microseismic degree of the lower limb of the rabbit. Rabbits of groups EA1 and EA2 were treated three times a week for 2 weeks. According to the modified Lequesne MG index score, the stimulation responses, including local pain, gait changes, range of motion, and joint swelling, were recorded before and after treatment, and the behavior of rabbits in each group was evaluated. Table 1 shows the specific standards of scoring.

**TABLE 1:** Lequensne Index of rabbits’ behavior.

Indices	Grade	Animal performance	Score
Local pain irritation	I	No abnormal pain response	0
	II	The affected limb shrinked	1
	III	The affected limb showed constriction and spasm, accompanied by mild systemic reactions such as turning back and sucking, and shaking the whole body, etc.	2
	IV	The affected limb showed violent contraction, tremor, spasm, struggle, and abnormal vocalization	3

Gait changes	I	The affected limb pushed the ground vigorously, without limping. Its running and moving were normal	0
	II	The affected limb pushed on the ground forcefully when it was running with a mild limp	1
	III	The affected limb was obviously limping	2
	IV	The affected limb could not push nor touch the ground nor participate in walking	3

Joint range of motion	I	Above 90°	0
	II	45°–90°	1
	III	15°–45°	2
	IV	Below 15°	3

Joint swelling	I	There was no swelling of the joints, and the bony marks are clearly clear	0
	II	The joints were slightly swollen and the bony marks became shallow	1
	III	Joint swelling was obvious and bony marks disappeared	2

### Specimen Collection

The specimens of the two model groups were collected after the modeling was accomplished, and the samples of the EA groups were collected after treatment. Before the specimens were collected, the experimental rabbits were prohibited from water for 12 h. They were then fixed and given anesthesia with sodium pentobarbital solution (3% concentration, 1 ml/kg). Touching the strongest position of the heart throb of the rabbit (between the third and fourth ribs), a 20-ml syringe was inserted into the heart at 30°–40° from the left side of the cartilage. When the tip trembled slightly, the syringe was gently withdrawn. About 10 ml of blood was collected and placed in a yellow biochemical tube to stand for 2 h. The upper serum was taken and placed in a centrifugal tube for shaking and centrifuged at 12,000 r/min for 10 min. The supernatant was taken, labeled, and stored in a refrigerator at –20°C for inspection. The rabbits were euthanized, and their knee joints were dissected after disinfection. The synovium and the articular cartilage were firstly observed by naked eyes. Then, the synovium, the proximal segment of the tibia, and the distal segment of the femur articular cartilage were cut off completely. The specimens were frozen in liquid nitrogen immediately and stored in a refrigerator at –80°C for testing. Some specimens of the cartilage and the synovium were fixed in 4% paraformaldehyde.

### Testing Indicators

#### Hematoxylin-Eosin Staining (HE Staining)

The cartilage and synovial tissues fixed in 4% paraformaldehyde were removed and pruned, placed in an embedding box, dehydrated with ethanol gradient, embedded in conventional paraffin, and sected. They were then stained with HE staining slides of the stained tissue were successively placed under a microscope for morphological observation and photographed for retention. According to the Kreen and Mankin scoring standards, the pathology of the synovium and the cartilage was calculated (Mankin et al., 1981; Krenn et al., 2002).

#### Enzyme-Linked Immunosorbent Test

The serum samples of each group were thawed at room temperature and shaken well. The contents of TNF-α, IL-1β, IL-6, MMP1, MMP3, and MMP13 in the serum of rabbits in each group were determined by ELISA according to the procedure of kit operation. Three wells were set for each index.

#### Protein Western Blot Analysis

After shearing and grinding, the 200 μL precooled RIPA protein lysate was added to the synovium and fully cracked on ice for 30 min. The centrifuge was performed at 15,000 rpm/min at 4°C for 30 min. After that, the supernatant was absorbed, placed into a new EP tube, and labeled. The BCA protein kit was used to measure the total protein concentration in each sample, and denaturation treatment was performed to keep the concentration of each histone consistent.

The protein molecules were then isolated on an SDS-PAGE gel and transferred to a polyvinylidene fluoride (PVDF) membrane, which was completely immersed in 3% BSATBST and gently shaken for 30 min at room temperature. The first antibody was diluted with 3% BSA-TBST and labeled according to the instructions of TLR2, TLR4, NF-κB, MyD88, TRAF6, and internal reference β-actin. Then, the samples were cultured with horseradish peroxidase (HRP) labeled as secondary antibody (Abcam, United States) and finally mixed with high-sensitivity ECL chemiluminescence reagent (Proteintech, United States).

#### Quantitative PCR Analysis

The messenger RNA (mRNA) expressions of TLR2, TLR4, NF-κB, MyD88, and TRAF6 in synovial tissues of each group were detected by qPCR. The primer sequence design of each component is shown in Table 2. According to the kit instructions, Trizol total RNA extraction reagent was used to extract RNA from the specimens. UV absorption method was used to detect the concentration and purity of synovial mRNA in each group, and agarosegel electrophoresis was used to detect the integrity of the mRNA. Gene expression was detected using a PCR kit and data using 2^–△△CT^ method to be analyzed.

**TABLE 2:** Primer sequence design of TLR4, NF-κB, MyD88, TLR2, and TRAF6.

Gene	Primer sequence (5′–3′)	
TLR4	F:GAAAGTATGGTAGGGG TGAAAGCG	R:GTGAAGGCAGAGCCG AAAGG
NF-κB	F:GAAGGACAAGACCAAA TTCTCAGT	R:GCAGGCTATTGCTCAACACG
TRAF6	F:ACTATTCAGCAGTT AGAGGGTCG	R:CCATTTTTGCAGTCAGCTCC
MyD88	F:CTACTGCCCCAGCGACAT	R:CTACTGCCCCAGCGACAT
TLR2	F:GGGCTGTCTGTCACCGAAC	R:GGATGTGCAACCTCCGTATT
ACTIN	F:AAGTGCGACGTGGACATCCG	R:GGGCGGTGATCTCCTTCTGC

### Statistical Analysis

SPSS22.0 is used for data analysis, and the measurement data were expressed by the mean ± SD (‘x ± S). If the data conformed to the normal distribution and the homogeneity of variance, the two groups of data were compared by *t*-test. One-way ANOVA was used for more than two groups. If one item was not consistent, then the rank-sum test of multiple independent samples in non-parametric statistics was used for statistical processing. The chi-squared test was used for grade data, with *p* < 0.05 as the standard of statistical difference.

## Results

### Behavior Observation

After the model group was modeled and the EA groups were EA treated, various indicators were evaluated and recorded according to the modified Lequesne MG index score. The results are shown in Table 3. As the table shows, Group S, Group M1/M2, and Group EA1/EA2, compared to Group B, showed statistically significant differences (*p* < 0.05). The behavioral scores of Group B were significantly lower than those of other groups. There is a significant difference between Group EA1 and Group M1 (*p* < 0.05) and a significant difference between Group EA2 and Group M2 (*p* < 0.05). These results show that EA significantly improved the behavioral function of the KOA rabbits.

**TABLE 3:** Statistical analysis of the Lequensne index of rabbit behavior in each group.

Groups	B	S	M1	M2	EA1	EA2
Indices	0.00 ± 0.00	5.50 ± 0.75*	7.38 ± 0.52*	8.38 ± 0.91*	3.15 ± 0.92*^#^	4.50 ± 0.53*^#^

*Indicates that there is statistically significant difference with the blank group, *P* < 0.05. ^#^Indicates that the difference between the Group M1 compared with Group EA1 is statistically significant, and the difference between Group M2 compared with Group EA2 is statistically significant *P* < 0.05.

### Histological Analysis

#### Hematoxylin and Eosin Staining of the Cartilage Tissue

**FIGURE 1:**
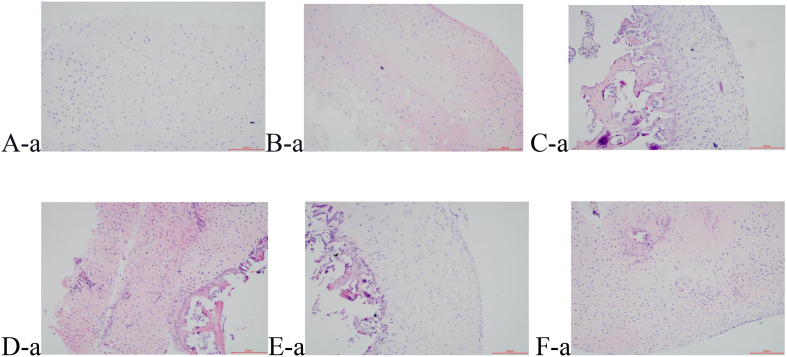
HE staining results of cartilage tissue, **(A-a)** Group B; **(B-a)** Group S; **(C-a)** Group M1; **(D-a)** Group M2; **(E-a)** Group EA1; **(F-a)** Group EA2.

H&E staining obviously shows that the pathological process of the cartilage gradually aggravated with time. The cartilage surface in groups B and S (A-a and B-a) was smooth and the cells were distributed regularly. The cartilage layer was also clearly stratified, and the tide line was intact. The nucleus staining was dark blue, and the cytoplasm and the cartilage matrix were uniformly pink. Compared with Group S, although Group M1 (C-a) showed a smooth surface and an irregular cell arrangement, the cartilage was stratified, and the tidal line was intact. In Group M2 (D-a), superficial ulcers appeared on the surface of the cartilage, and many cracks perpendicular to the surface were seen in the deep matrix part of the cartilage. The cell sizes were different, with the contour being vague, some cells being necrotic, the thickness of the cartilage significantly reduced, the tide line disorganized, and the calcification layer thickened. Group EA1 and Group EA2 (E-a and F-a) showed less clear contour, and their cells were less regularly distributed. The tidal line was slightly broken and showed less clear stratification. The above results are shown in Figure 1. The cartilage tissue was scored using the modified Mankin pathological scoring standard. The results are shown in Table 4. The difference between the other groups and the blank group was significant. Groups M1 and M2 were scored 7.00 and 9.25, respectively, while Group M1 was significantly lower than Group M2. Groups EA1 and EA2 were scored 4.13 and 5.75, respectively, indicating that they were both lower than that of their corresponding model groups.

**TABLE 4:** Rabbit cartilage histology Mankin Grading Standard.

Groups	B	S	M1	M2	EA1	EA2
Indices	0.63 ± 0.18	0.89 ± 0.0.23	7.00 ± 0.50*	9.25 ± 0.49*	4.13 ± 0.29*^#^	5.75 ± 0.31*^#^

*Indicates that there is statistically significant difference with the blank group, *P* < 0.05. ^#^Indicates that the difference between Group M1 compared with Group EA1 is statistically significant, and the difference between Group M2 and Group EA2 is statistically significant, *P* < 0.05.

#### Hematoxylin and Eosin Staining of the Synovial Tissue

**FIGURE 2:**
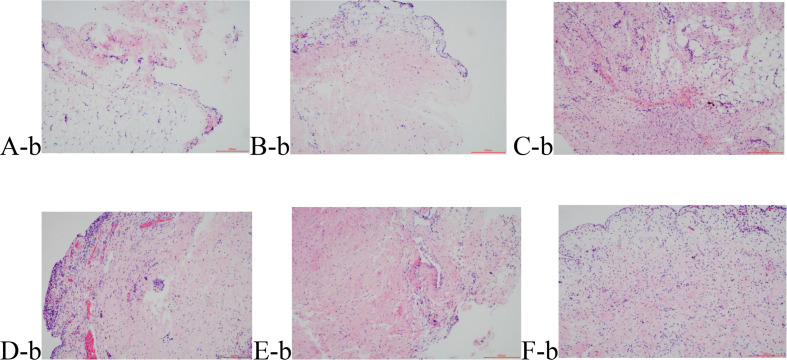
Hematoxylin eosin (HE) staining results of the synovial tissue. **(A-b)** Group B; **(B-b)** Group S; **(C-b)** Group M1; **(D-b)** Group M2; **(E-b)** Group EA1; and **(F-b)** Group EA2.

The normal synovial tissue structure is composed of one or two layers of synovial cells arranged in a tile form. H&E staining showed that the synovial lining cells in Group B were distributed in a monolayer or double-layer structure without hyperplasia or hypertrophy (Figure 2A-b). Morphological distribution of the synovial cells in Group S was similar to Group B, without either hyperplasia or pannus formation (Figure 2B-b), which validated that the operation method did not have an effect on the model lesions. Compared with Groups B and S, Group M1 (Figure 2C-b) showed hyperemia in the synovial tissue, obvious inflammatory infiltration of focal lymphatic, mononuclear, and plasma cells, dense fibrosis in the subsynovial tissue, and hyperplasia of the capillaries. Group M2 (Figure 2D-b) showed not only obvious inflammatory infiltration of cells but also papillary hyperplasia on the synovial surface, Synovial cells significantly reduced in number, and the cells became atrophied. The synovial hyperplasia and lymphocyte infiltration in Groups EA1 and EA2 (Figures 2E-b,F-b) were significantly less severe than those in the corresponding model group. The results are shown in Figure 2. Krenn pathological scale was used to score the degree of synovial tissue lesions, and the results are shown in Table 5. The difference between the other groups and the blank group was significant. The mean scores of the cartilage tissue in groups M1 and M2 were 3.75 and 6.33, respectively, showing that Group M1 scored significantly lower than Group M2. The mean scores of the cartilage tissue in groups EA1 and EA2 were 2.50 and 3.87, respectively, and both lower than those of their corresponding model group.

**TABLE 5:** Rabbit synovial histology Krenn Grading Standard.

Groups	B	S	M1	M2	EA1	EA2
Indices	0.50 ± 0.19	0.75 ± 0.16	3.75 ± 0.41*	6.63 ± 0.32*	2.50 ± 0.26*^#^	3.87 ± 0.39*^#^

*Indicates that there is statistically significant difference with the blank group, *P* < 0.05. ^#^Indicates that the difference between Group M1 compared with GGroup EA1 is statistically significant,and the difference between Group M2 and Group EA2 is statistically significant, *P* < 0.05.

#### Enzyme-Linked Immunosorbent Assay

**FIGURE 3:**
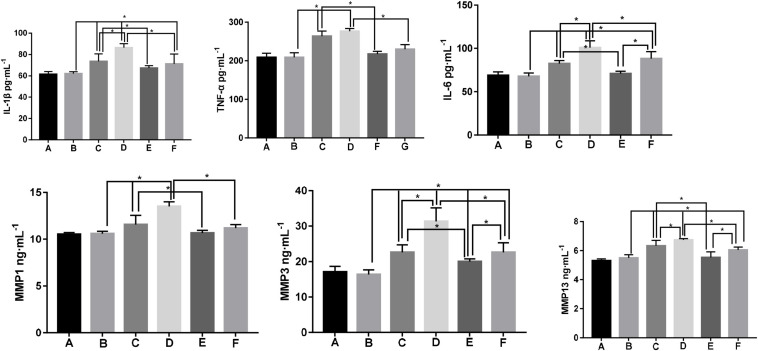
The expression of TNF-α, IL-1β, IL-6, MMP1, MMP3, and MMP13 in the serum of rabbits of each group. *Note* (A) Group B; (B) Group S; (C) Group M1; (D) Group M2; (E) Group EA1; and (F) Group EA2. *P < 0.05.

The contents of TNF-α, IL-1β, IL-6, MMP1, MMP3, and MMP13 in the serum of each group were detected by ELISA. The results show that there was no significant difference in the contents of TNF-α, IL-1β, IL-6, and MMP1, MMP3, and MMP13 between the serum of Group B and those of Group S (*p* > 0.05), which could help us rule out the interference from the operation. The contents of the inflammatory factors and the matrix metalloproteinases were significantly different from those of groups M1 and M2 (*p* < 0.05) and increased gradually with the modeling prolonged. Compared with groups M1 (*p* < 0.05) and M2 (*p* < 0.05), the contents of TNF-α, IL-1β, IL-6, MMP1, MMP3, and MMP13 in the EA groups significantly decreased, indicating that the expression of the inflammatory factors and matrix metalloproteinases was significantly inhibited after the EA treatment. However, the levels of TNFα, IL-6, and MMP3 in Group EA1 were significantly lower than those in Group EA2 (*p* < 0.05), which indicates that early EA intervention could better alleviate the inflammation. The results are shown in Figure 3.

### Western Blot Analysis of Protein

**FIGURE 4:**
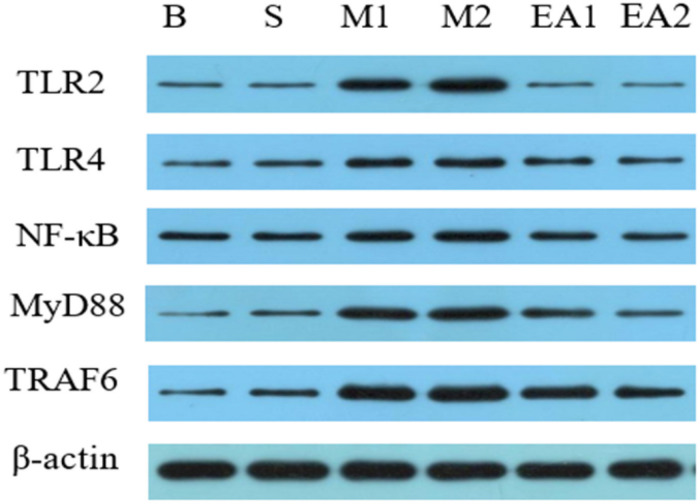
Western blot detection of synovial TLR2, TLR4, NF-κB, MyD88, and TRAF6 protein expression Western blot.

**FIGURE 5:**
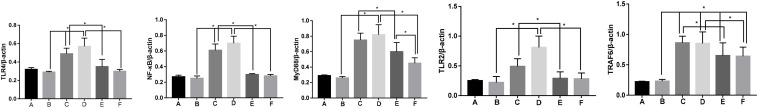
Protein expression levels of TLR4, NF-κB, MyD88, TLR2, and TRAF6 in the synovium of rabbit joints in each group. *Note* (A) Group B; (B) Group S; (C) Group M1; (D) Group M2; (E) Group EA1; and (F) Group EA2. *P < 0.05.

The expressions of TLR2, TLR4, NF-κB, MyD88, and TRAF6 protein in the synovial tissue of each group were detected by Western blot, as shown in Figures 4, 5. The expressions of TLR2, TLR4, NF-κB, MyD88, and TRAF6 protein in the synovium of Groups M1 and M2 were significantly higher than those of Group S (*p* < 0.01) whereas the expressions of TLR2, TLR4, NF-κB, MyD88, and TRAF6 protein in the synovial tissue after EA treatment were significantly lower than those of corresponding groups M1 and M2 (*p* < 0.01). The protein expression of Group M2 was higher than that of Group M1 (*p* < 0.05). There was a significant difference in the expression of protein in MyD88 between Group EV1 and EV2 (*p* < 0.05). However, there was no significant difference in protein expressions of TLR4, NF-κB, TRAF6, and TLR2 (*p* > 0.05) between groups EV1 and EV2.

### Analysis of qPCR Results

**FIGURE 6:**
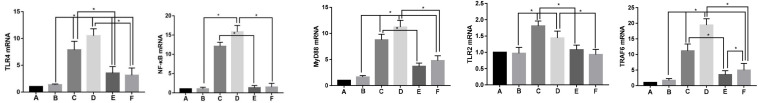
mRNA expression levels of TLR2, TLR4, NF-κB, MyD88, and TRAF6 in the synovium of rabbit joints in each group. *Note* (A) Group B; (B) Group S; (C) Group M1; (D) Group M2; (E) Group EA1; and (F) Group EA2. *P < 0.05.

The mRNA expressions of TLR2, TLR4, NF-κB, MyD88, and TRAF6 in the synovial samples of each group were detected by qPCR, as shown in Figure 6. The mRNA expression levels of TLR2, TLR4, NF-κB, MyD88, and TRAF6 in the synovium of groups M1 and M2 were significantly higher than those of Group S (*p* < 0.05), whereas the mRNA expression of Group M2 was higher than that of Group M1 (*p* < 0.05). mRNA expression levels of TLR2, TLR4, NF-κB, MyD88, and TRAF6 in the synovial tissue after the EA treatment were significantly lower than those in their corresponding groups M1 and M2 (*p* < 0.05). The mRNA expressions of MyD88 and TRAF6 in groups EV1 and EV2 were significantly different from each other (*p* < 0.05), and the expressions in Group EV2 were higher than those in Group EV1. However, the difference in the mRNA expressions of TLR2, NF-κB, and TLR4 between groups EV1 and EV2 was not statistically significant (*p* > 0.05).

## Discussion

To our knowledge, synovitis plays an important role in the pathological and clinical manifestations of KOA. During the disease development in many patients with KOA, synovitis of the joint is characterized by non-infectious chronic inflammation (Band et al., 2015). These pathological manifestations have been confirmed by our morphological research results. Synovial pathological H&E dyeing can be observed, with the increase in the severity of synovium lesions, from the normal articular synovial to mild hyperemia of synovial tissue, focal inflammatory infiltration of lymphocytes, and plasma cells, and then to the clearly thickened hyperplastic synovial, increased amount of fibrous tissue, capillary sclerosis involvement, showing varying degrees of performance.

In addition, many studies have shown that arthroscopy, MRI, and ultrasound can detect the degree of synovitis progression, which can be used as an indicator to evaluate the severity of OA lesions. Synovitis is reported to be correlated with the imaging grade of OA (Roemer et al., 2011; Neogi, 2017). Synovial lesions accompany the KOA occurrence and development throughout the whole pathological process, from early inflammation to synovial hyperplasia, from the production of inflammatory mediators to the destruction of the cartilage. They play an important role because they get various immune cells, cytokines, and signaling pathways involved in this pathological process. To sum up, as the focus of research and treatment of KOA merely on chondro-pathies has shifted, a multi-directional approach may help break through the bottleneck in KOA treatment. At present, nevertheless, there is still a controversy over the cause of KOA synovial inflammation.

The theory of synovial innate immune response, as a mechanism that has been relatively recognized as regards the cause in question, is a decisive defense line of the body against the invasion of various pathogens (Miller et al., 2019). Due to the degeneration of the joint, the destroyed extracellular matrix and the shed debris enter the joint cavity, stimulate the synovium to initiate the innate immune response, and promote the release of inflammatory mediators into the synovial fluid. This can in turn stimulate the synovium and the cartilage to form an inflammatory response and then further damage the intra-articular environment (Berenbaum, 2013). TLRs are one of the most important PRRs in the innate immune response, and their mediated innate immune response to synovitis is the key link in promoting KOA synovitis. The TLR activates transcription factor NF-κB by participating in recognition of pathogen-associated molecular pattern molecules (PAMPs) and perception of damage-associated molecular patterns (DAMPs) and then activates transcription and expression of a series of pro-inflammatory factors such as IL-1β (Fitzgerald and Kagan, 2020). IL-lβ is released to the outside of the cell by activating the IL-1 signaling pathway and the MyD88/NF-κB signaling pathway, recruits inflammatory cells, stimulates the synovial cell proliferation, promotes angiogenesis, and releases more inflammatory factors and protease into the joint. Consequently, the inflammation cascade effect is amplified, with the OA inflammation sustained, and the OA disease process further aggravated (Sidiropoulos et al., 2008). Pathogenic microorganisms, known as PAMPs, are a classical ligand for the activation of TLRs. However, studies have shown that even in the absence of pathogenic microorganisms, TLRs can be activated by DAMPs to initiate an innate immune response (van den Bosch, 2019). DAMPs include endogenous substances distributed in OA synovial fluid such as small molecular weight hyaluronic acid, fibronectin, cytokinin C, and high-mobility group B1 (HMGB-1) (Scanzello et al., 2008; García-Arnandis et al., 2010; van Lent et al., 2012). These endogenous substances are caused by the destruction of the extracellular matrix due to aseptic tissue injury or cellular stress (Piccinini and Midwood, 2010). At present, the role of TLRs in inflammatory and rheumatic diseases such as rheumatoid arthritis (RA), systemic lupus erythematosus (SLE), and tissue damage has been well established. Therefore, the pathogenesis of KOA probably lies in the fact that the synovial innate immune response activates the synovial inflammatory response of OA through DAMPs/TLRs/NF-κB signaling pathway.

Electroacupuncture therapy, developed based upon acupuncture and moxibustion, is a physical therapy that combines acupuncture with electric pulses. It is multi-layered, multi-channeled, and multi-targeted. The mechanism may be that the endocrine system releases hormones, the autonomic nervous system releases neurotransmitters, and the signal transduction pathway regulates the secretion of cytokines and the immune response of the body, thus effectively alleviating the clinical symptoms of patients (Kwon et al., 2006; Wang et al., 2008; Mata et al., 2015). Adjusting the current intensity, frequency, and action duration of acupoints can enhance the acupuncture induction and improve the local microcirculation, dredge local meridians, and achieve analgesic effects. There have been many studies recently on EA analgesia. For example, it has been reported that for the treatment of KOA, EA is not only analgesic but it can also reduce local inflammatory responses and improve microcirculation. Results of this study show that EA can better mitigate the soft tissue tension by stimulating the local acupoints, relieve the positive reaction points of the junction around the knee, and can thus thin and soften the tendons, dredge the collaterals, and relieve KOA-related pains.

Seo et al. (2013) reported that low-frequency (2 Hz) EA at Zusanli had a good analgesic effect on collagenase-induced KOA rats. Qin et al. (2013) found that EA treatment of New Zealand white rabbits with knee arthritis model induced by bilateral ovariectomies resulted in higher estrogen levels in the EA group than in the model group, thereby reducing MMP-13 expression. So far, despite that the mechanism of EA in improving knee arthritis has not been fully elucidated, recent studies have reported that EA may have an anti-inflammatory effect on knee arthritis (Chen et al., 2017; Sun et al., 2018; Wu et al., 2019).

This study is intended to elucidate the mechanism in question. We used the modified Lequesne MG to observe the behavioral changes of rabbits of each group. The profile score of the model group was significantly higher than that of the blank group, indicating that the model was successfully established. The improved Hulth method resulted in limited knee function of the model rabbits. The behavioral records of each group before and after the treatment showed that EA could significantly improve the knee function of the model rabbits and decrease their behavioral score. Morphological analysis shows that there were obvious differences in each rabbit cartilage and synovial membrane and that the severity of the damage to the cartilage and synovial membrane gradually increased with the modeling prolonged. This complies with the research results of the study by Wang (2018), who believed that the degree of synovial inflammation increased gradually with the modeling prolonged in the early and middle stages, while the degree of inflammation decreased in the late stages, indicating that synovial inflammation had lesions in early KOA. In addition, as EA is a conservative treatment, this study selected the early and middle stages of KOA to test the intervention effect of EA on KOA treatment. After the treatment, it was found that the inflammatory state of the synovial membrane and the degree of cartilage injury were alleviated in groups EA1 and EA2 in comparison with those in the corresponding Groups M1 and M2. This is clearly demonstrated by the pathological scores of the synovium and the soft tissue, indicating that EA can play a curative role in KOA treatment.

It is accepted that the theory of innate immune response may explain the mechanism of synovitis. Nevertheless, it remains to be verified whether EA plays a therapeutic role by regulating the innate immune response. Our ELISA results have confirmed that the contents and levels of TNF-α, IL-1β, IL-6, MMP1, MMP3, and MMP13 in the serum of Group M2 were higher than those of Group M1, which agrees with the morphological results of this study. With modeling time extended, synovial inflammation gradually deteriorated. In other words, the longer the modeling, the more severe the synovial inflammation would be. The levels of TNF-α, IL-1β, IL-6, MMP1, MMP3, and MMP13 in groups EV1 and EV2 are significantly lower than those in their corresponding groups M1 and M2, which indicate that the expression of inflammatory factors and matrix metalloproteinases were significantly inhibited by EA treatment. In addition, the result that the levels of TNF-α, IL-6, and MMP3 in Group EV1 are significantly lower than those in Group EV2 indicating that early EA intervention can better restrain the inflammatory level. The results of Western Blot confirmed that the expression of TLR2, TLR4, and NF-κB protein in the synovium of Group M2 was higher than that of Group M1, whereas the expression of TLR2, TLR4, NF-κB, MyD88, and TRAF6 in synovial tissue after EA was significantly lower than those in corresponding groups M1 and M2. The qPCR results confirmed that the mRNA contents of TLR2, TLR4, NF-κB, MyD88, and TRAF6 in Group M2 were significantly higher than those in Group M1, whereas the expression of TLR2, TLR4, NF-κB, MyD88, and TRAF6 in synovial tissue after EA was significantly lower than those in corresponding groups M1 and M2. The mRNA expression level of MyD88 and TRAF6 in Group EV1 was lower than that in Group EV2.

From the aforementioned results, we may conclude that electroacupuncture treatment can alleviate knee OA by inhibiting the innate synovial immune response mediated by TLRs, reducing the expression of TLRs/NF-kb signal through relevant elements, and restraining the release of downstream inflammatory factors and matrix metalloproteinases. In addition, early intervention by electroacupuncture treatment of KOA lesions may have a better curative effect than in other stages of the disease.

## Data Availability Statement

The original contributions presented in the study are included in the article/supplementary material, further inquiries can be directed to the corresponding author.

## Ethics Statement

This study was approved by the Medical Ethics Committee of Beijing University of Chinese Medicine Third Affiliated Hospital (BZYSY-2019KYKTPJ-26).

## Author Contributions

YY, AR, and QW jointly completed the experimental design. YM and LY assisted AR in animal feeding, intervention, and sampling. AR, QX, DZ, and ZW carried out sample testing and data processing. YY and AR conceptually designed the study and prepared the manuscript. All authors contributed to the article and approved the submitted version.

## Conflict of Interest

The authors declare that the research was conducted in the absence of any commercial or financial relationships that could be construed as a potential conflict of interest.

## Publisher’s Note

All claims expressed in this article are solely those of the authors and do not necessarily represent those of their affiliated organizations, or those of the publisher, the editors and the reviewers. Any product that may be evaluated in this article, or claim that may be made by its manufacturer, is not guaranteed or endorsed by the publisher.
